# Expression of CASC8 RNA in Human Pancreatic Cancer Cell Lines

**DOI:** 10.1134/S1607672922040020

**Published:** 2022-08-29

**Authors:** O. Y. Burenina, N. L. Lazarevich, I. F. Kustova, T. S. Zatsepin, M. P. Rubtsova, O. A. Dontsova

**Affiliations:** 1 Center for Molecular and Cellular Biology, Skolkovo Institute of Science and Technology, Moscow, Russia; 2 Institute of Carcinogenesis, Blokhin National Medical Research Center of Oncology, Ministry of Health of the Russian Federation, Moscow, Russia; 3 Biology Department, Moscow State University, Moscow, Russia; 4 Chemistry Department and Belozersky Institute of Physico-Chemical Biology, Moscow State University, Moscow, Russia

**Keywords:** long noncoding RNAs, CASC8, pancreatic cancer

## Abstract

A lot of long non-coding RNAs (lncRNAs) are expressed in human cells in a number of transcripts of different lengths and composition of exons. In case of cancer-associated lncRNAs, an actual task is to determine their specific isoforms, since each transcript can perform its own function in carcinogenesis and might have a unique expression profile in various types of tumors. For the first time, we analyzed the expression of CASC8 lncRNA in human pancreatic ductal adenocarcinoma cell lines and found an abundant isoform that was previously considered as the minor one in this type of cancer. We also revealed extremely high expression levels of all CASC8 transcripts in MIA PaCa-2 cells and, conversely, the lack of this lncRNA in PANC-1. This allows to use them as convenient models for further in vitro studies.

Pancreatic cancer (predominantly pancreatic ductal adenocarcinoma, PDAC) is the most lethal among malignant neoplasms. Almost asymptomatic development and late detection of the tumor cause extremely low patient survival, which does not exceed 7% on average over 5 years, and the recurrence rate after surgery or chemotherapy is extremely high [1]. To date, only the CA 19-9 oncomarker, which is determined in blood serum, is used in clinical practice to diagnose and predict PDAC development. However, the scope of its application is extremely limited due to low sensitivity and specificity, since its increase is also observed in other tumors of the digestive system, as well as in non-tumor pathologies [2]. A promising direction is the use of long non-coding RNAs (lncRNAs) as tumor markers, which can be directly measured in biological fluids, biopsy specimens, or postoperative tissues using real-time PCR with reverse transcription (RT-PCR) [3]. To date, dozens of lncRNAs, which expression levels change during PDAC, are known [4, 5]. However, the majority of them, such as MALAT1, ANRIL, H19, UCA1, etc., are “universal” oncogenic lncRNAs. The lncRNAs that are more tissue-specific for the pancreas or for the digestive system in general usually have low expression levels, which limits the possibility of their use in clinical practice and impedes studies on elucidation of their properties and functions. Thus, identification of new lncRNAs which expression is activated in PDAC in comparison to healthy pancreatic tissues is a relevant task.

In the course of a preliminary analysis of the available bioinformatic data from TCGA (The Cancer Genome Atlas), we found a miserably studied lncRNA CASC8 (aka “Cancer Susceptibility Candidate 8”). Its expression was significantly activated in cancers of the gastrointestinal tract and lungs (Fig. 1a) with the maximum in the case of PDAC. At the same time in normal pancreatic tissues this lncRNA was almost absent. These differences were statistically significant, which makes possible predicting the diagnostic potential of CASC8 (Fig. 1a). The high mortality rate of patients with elevated levels of CASC8 expression (Fig. 1b) indicates its probable pro-oncogenic role and allows considering this lncRNA as a potential prognostic biomarker.

**Fig. 1.**
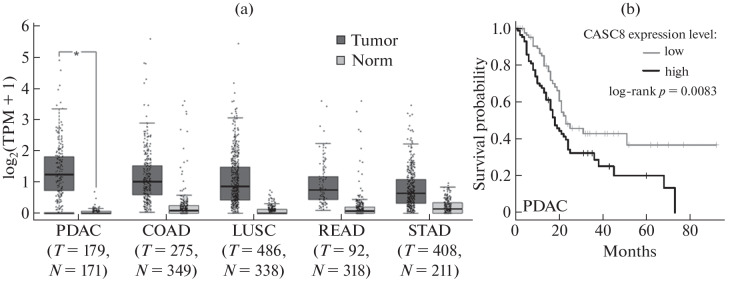
CASC8 expression in various types of cancer according to the GEPIA online server (Gene Expression Profiling Interactive Analysis, www.gepia.cancer-pku.cn). (a) Top 5 tumors with the maximum CASC8 expression compared to non-tumor tissues. Averaged TCGA data for the number of specimens. Designations: T—tumor, N—norm, PDAC—pancreatic ductal adenocarcinoma, COAD—colon adenocarcinoma, LUSC—lung squamous cell carcinoma, READ—rectal adenocarcinoma, STAD— stomach adenocarcinoma. (b) Overall survival (Kaplan–Meier curve) of patients with PDAC in groups with high (*n* = 88) and low (*n* = 88) CASC8 expression levels in postoperative tumor samples.

However, most of the published studies on CASC8 were made from analytical point of view and were devoted to establishing the correlation of individual nucleotide polymorphic substitutions (SNP) in its gene with the risks of development of various types of cancer [6]. Attempts to reveal the functional role of the CASC8 lncRNA itself were made only in several experimental studies. It was shown that CASC8 expression in retinoblastoma cells promoted a decrease in the level of tumor suppressor miR-34a and subsequent increase in proliferative activity [7]. In the case of non-small cell lung cancer, decrease of CASC8 expression suppressed the ability of cells to proliferate, invade, migrate, and form colonies and increased their sensitivity to osimertinib [8]. In [9], it was demonstrated that the suppression of CASC8 expression in PDAC leads to the activation of miR-129-5p and the inhibition of TOB1 mRNA; however, the biological significance of this effect, as well as the function of TOB1 in carcinogenesis, has not been elucidated.

We found that all studies completely ignored the existence of four different CASC8 isoforms annotated in databases (Ensembl, NCBI, USCS). Moreover, in 2014, the 5'- and 3'-ends of all CASC8 transcripts were determined in colon cancer cells in experiments performed by the RACE (“Rapid Amplification of cDNA Ends”) method [10]. On the basis of the obtained results (Fig. 2a), it can be concluded that *CASC8* gene expression products are represented by a long transcript (*i1*), which contains the first three exons, and two short isoforms: *i2* contains exons 5, 6, and 7, whereas *i3* contains exons 5 and 7. According to [10], the *i1a* isoform is not expressed and is probably the result of annotation of the lncRNA precursor. The transcripts *i1* and *i2*/*i3* do not have common exons, i.e., they are two completely different lncRNAs. It is noteworthy that, in all previously published studies on CASC8, primers complementary to exons 1–3 (i.e., specific to *i1*) were used in RT-PCR. However, according to the available information from the UCSC database, this isoform is almost absent in PDAC tissues. Thus, the aim of this study was to identify different CASC8 lncRNA isoforms in PDAC cell cultures and to compare their expression levels.

**Fig. 2.**
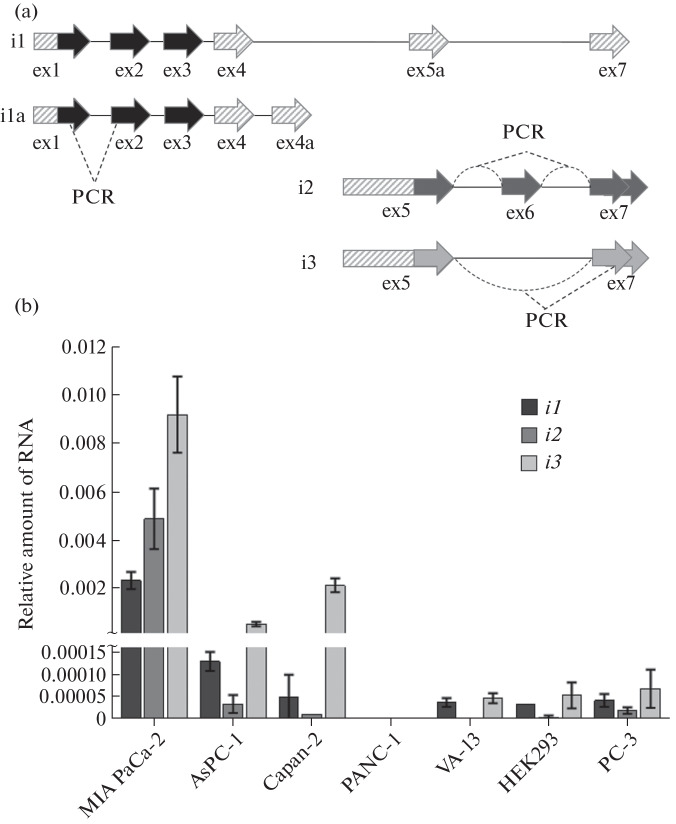
Comparative analysis of relative expression (RT-PCR) of different CASC8 isoforms. (a) Scheme of exon organization of CASC8 transcripts. Hatching denotes the exons (or their parts) that are annotated in databases, and solid color denotes the exons whose presence was confirmed experimentally [10]. The dotted lines indicate the regions complementary to the PCR primers used for the amplification of each isoform. (b) Expression levels of CASC8 transcripts (*i1, i2*, and *i3*) in various pancreatic cancer cell lines compared to the control cells.

Four lines of pancreatic ductal adenocarcinoma were selected as study objects: MIA PaCa-2, PANC-1, AsPC-1, and Capan-2, which differ in the proliferation rate and/or differentiation grade [11]. PC-3 (prostate adenocarcinoma), VA-13 (lung fibroblasts) and HEK293 (embryonic kidney) cells were also used as controls. The amount of RNA was measured by RT-PCR, and U6 snRNA was used for normalization. The pairs of primers for PCR were selected in the way to be complementary to different CASC8 exons, and in the case of *i2* and *i3* they were complementary to exon junctions (Fig. 2a); thus, each isoform was amplified independently.

All three control lines PC-3, VA-13, and HEK293, as well as Capan-2 (the least aggressive PDAC line), showed extremely low expression levels of the CASC8 *i1* transcript, which was barely above the RT-PCR detection level. A slightly more pronounced expression was detected in the case of AsPC-1, whereas the amount of *i1* for MIA PaCa-2 was ~20 times higher. In addition, an extremely high level of *i2* expression was also detected in these cells, although, according to the distribution of transcripts in other analyzed cell lines, *i2* is a minor isoform. Conversely, the *i3* isoform apparently is predominant in PDAC cells, and its maximum expression was also detected in MIA PaCa-2.

PANC-1 was an exception as far as none of the CASC8 transcripts were detected. Thus, this cell line is a peculiar natural knockdown, which can be used in subsequent experiments. In contrast, MIA PaCa-2 cells express the maximum amount of all CASC8 RNA variants, including the conventionally minor transcript *i2*. We suggest this difference originates from the different nature of MIA PaCa-2 and PANC-1 [12]. Although both cell lines derive from aggressive poorly differentiated PDAC tumors, they are quite different in morphology, cell population heterogeneity, and expression of various factors. The main controversy is the epithelial–mesenchymal transition (EMT) status of these lines. Although both MIA PaCa-2 and PANC-1 exhibit the so-called mesenchymal phenotype, in most scientific literature they are considered a “quasi-mesenchymal” subtype of PDAC cells [13]. It should be noted that PANC-1 almost does not express E-cadherin and has a more pronounced metastatic potential [12]. Nevertheless, in the AsPC-1 and Capan-2 cell lines, *i3* is also the predominant CASC8 isoform. This indicates the limited applicability of the currently published data on the functioning of CASC8 in PDAC [9], because only the *i1* isoform was considered in these studies. We suggest that incorrect annotation of CASC8 isoforms may also be the reason why some scientific studies failed to confirm the activation of this lncRNA in the pancreatic tissues of patients with PDAC [14], since primers complementary to *i1* were used in RT-PCR. Correct annotation of the major CASC8 transcript in subsequent studies is also necessary for evaluation of its diagnostic potential.

Thus, this is the first study that evaluated the expression of various CASC8 isoforms in pancreatic ductal cancer cells and identified the most abundant transcript. We found that the maximum expression of all CASC8 isoforms is observed in the MIA PaCA-2 cell line, whereas in PANC-1 cells this lncRNA is not expressed at all. This difference makes it possible to use PANC-1 as a control line for in vitro experiments and a convenient model object for CASC8 overexpression. Notably, the *CASC8* gene is located at the 8q24.21 locus, that encodes a number of important oncogenic lncRNAs (PCAT1, PVT1, CCAT1, CCAT2, CCBC26, etc.) and microRNAs (miR-1204, miR-3686, miR-5194, etc.), which are located in close proximity to the *Myc* oncogene and affect its expression [15]. Elucidating the functional role of CASC8 (and its individual transcripts) may significantly contribute to understanding the mechanisms of regulation of this locus.
