# Erratum to: **Expression of CASC8 RNA in Human Pancreatic Cancer Cell Lines**

**DOI:** 10.1134/S1607672955340012

**Published:** 2023-02-14

**Authors:** O. Y. Burenina, N. L. Lazarevich, I. F. Kustova, T. S. Zatsepin, M. P. Rubtsova, O. A. Dontsova

**Affiliations:** 1 Center of Molecular and Cellular Biology, Skolkovo Institute of Science and Technology, Moscow, Russia; 2 Institute of Carcinogenesis, Blokhin National Medical Research Center of Oncology, Ministry of Health of the Russian Federation, Moscow, Russia; 3 Biology Department, Moscow State University, Moscow, Russia; 4 Chemistry Department and Belozersky Institute of Physico-Chemical Biology, Moscow State University, Moscow, Russia

The article “Expression of CASC8 RNA in Human Pancreatic Cancer Cell Lines,” written by O. Y. Burenina, N. L. Lazarevich, I. F. Kustova, T. S. Zatsepin, M. P. Rubtsova, and Academician O. A. Dontsova, was originally published electronically in Springer-Link on August 29, 2022 without Open Access. After publication in volume 505, issue 1, pages 137–140 the authors decided to make the article an Open Access publication. Therefore, the copyright of the article has been changed to © The Author(s) 2022 and the article is forthwith distributed under the terms of a Creative Commons Attribution 4.0 International License (http://creativecommons.org/licenses/by/4.0/, CC BY), which permits use, duplication, adaptation, distribution and reproduction of a work in any medium or format, as long as you cite the original author(s) and publication source, provide a link to the Creative Commons license, and indicate if changes were made.

